# Survival analysis of time to cure on multi-drug resistance tuberculosis patients in Amhara region, Ethiopia

**DOI:** 10.1186/s12889-019-6500-3

**Published:** 2019-02-07

**Authors:** Yigzaw Alemu Limenih, Demeke Lakew Workie

**Affiliations:** 1 Ethiopia Statistical Agency, Bahir Dar Branch, Bahir Dar, Ethiopia; 2 Statistics Department, Bahir Dar University, Bahir Dar, Ethiopia

**Keywords:** Amhara region, Multidrug-resistance tuberculosis, Average recovery time, Accelerated failure time models, Parametric shared frailty

## Abstract

**Background:**

Multidrug-resistant tuberculosis (MDR-TB) is caused by bacteria that are resistant to the most effective anti-tuberculosis drug. The MDR-TB is an increasing global problem and the spread of MDR-TB has different recovery time for different patients. Therefore, this study aimed to investigate the recovery time of MDR-TB patients in Amhara region, Ethiopia.

**Method:**

A retrospective study was carried out in seven hospitals having MDR-TB treatment center of Amhara region, Ethiopia from September 2015 to February 2018. An accelerated failure time and parametric shared frailty models were employed.

**Results:**

The study revealed that the recovery time of MDR-TB patients in Amhara region was 21 months. Out of the total MDR-TB patients, 110 (35.4%) censored and 201 (64.6%) cured of MDR-TB. The clustering effect of frailty model was hospitals and the Weibull-gamma shared frailty model was selected among all and hence used for this study. The study showed that extra pulmonary MDR-TB patients had longer recovery time than that of seamier pulmonary MDR-TB patients in Amhara region, Ethiopia. According to this study, male MDR-TB patients, MDR-TB patients with co-morbidity and clinical complication were experiencing longer recovery time than that of the counter groups. This study also showed that MDR-TB patients with poor adherence had longer recovery time than those with good adherence MDR-TB patients.

**Conclusion:**

Among different factors considered in this study, MDR-TB type, clinical complication, adherence, co-morbidities, sex, and smoking status had a significant effect on recovery time of MDR-TB patients in Amhara region, Ethiopia.

In conclusion, the Regional and Federal Government of Ethiopia should take immediate steps to address causes of recovery time of MDR-TB patients in Amhara region through encouraging adherence, early case detection, and proper handling of drug-susceptibility according to WHO guidelines.

## Background

Tuberculosis (TB) is the ninth leading cause of death worldwide and the leading cause of a single infectious agent, ranking above HIV/AIDS [1]. An estimated 10.4 million people had TB in 2016: 74% were in Africa and 56% were in India, Indonesia, China, the Philippines and Pakistan [1]. Globally in 2016, 6.6 million people with tuberculosis (TB) of these, just over 6.3 million had new or relapsed TB [1].

According to WHO multidrug-resistant tuberculosis (MDR-TB) is caused by bacteria that are resistant to the most effective anti-tuberculosis drugs (isoniazid and rifampicin) [1–3]. MDR-TB results either from primary infection or develop in the course of treatment of a patient due to human error, poor supply management, poor quality anti-TB drugs and/or improper treatment [2, 4, 5]. In addition, poor infection control practice has also been identified as a major factor for the spread of MDR-TB and MDR-TB has different recovery time for different patients [6]. MDR-TB is being an increasing global problem, and in 2016, 153,119 cases were notified from which 129,689 enrolled for treatment, of which only 22% started treatment [1]. Assefa et al also noted that 3.7% new and 20% previously treated MDR-TB cases were identified [7].

The burden and incidence of MDR-TB is increasing and varying significantly from country to country. The countries with the largest number of MDR/RR-TB cases (47% of the global total) were China, India and the Russian Federation [1]. The highest (28%) rate of new MDR-TB cases are from the Soviet Union including regions that share borders with the European Union [8]. In Africa, an estimated 69,000 cases emerged of which about 1.2% were new. 12% of re-treatment cases were from Ethiopia of which 1.6 and 12% MDR-TB patients were new and previously treated TB cases respectively [9]. In addition, Ethiopia is one among the 20 countries with the highest absolute estimated number of incidents of TB and MDR-TB [1]. In comparison to drug-susceptible TB, that takes about 6 to 9 months to treat, recommended treatment for MDR-TB lasts 18 to 24 months or longer [10], and requires the second line medicines that are not effective as first-line medicines commonly prescribed to treat TB [10]. Previous studies indicated that drug-resistant strains of *Mycobacterium tuberculosis* are of great concern as they are more toxic and more expensive than the first-line regimen [11]. Hence, monitoring closely patients while they take these drugs is critical, as the medications may lead to other serious health problems such as damage to the kidneys, liver, or heart; loss of vision or hearing; and changes in behavior or mood including depression or psychosis [12].

As Ethiopia is one of the 20 high burdens MDR-TB countries and MDR-TB has been a major health problem of the society in the Amhara region, a strategy to provide culture and drug susceptibility testing services has been designed [2, 7]. Even though various studies on the prevention and control of the cross-transmission of healthcare-acquired infections between hospitalized patients have been carried out, the prevalence is still increasing [13, 14]. Importantly, the appearance and transmission of MDR-TB is increasing in hospitals worldwide [15]. MDR-TB poses therapeutic difficulties in the twenty-first century, with only a few antibiotics continuing effective [16]. Consequently, controlling and preventing the emergence and overflow of MDR-TB organisms is of vital importance. Thus, the aim of this study is to investigate the recovery time of MDR-TB patients in Amhara region, Ethiopia using accelerated failure time and parametric shared frailty models.

## Method

### Data source, sampling design, and sample size

**Fig. 1:**
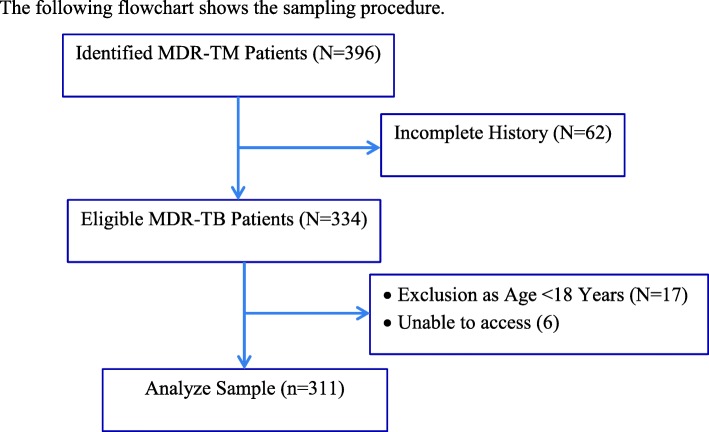
A Flow chart for sample selection

A retrospective study is carried out in seven hospitals of Amhara region which have MDR-TB treatment center from September 2015 to February 2018. Amhara National regional state is the second largest region of Ethiopia with an estimated area of 159,173.66 km^2^ and an estimated population density of 108.2 [17]. The region is divided into 11 administrative Zones, and 151 Woredas (128 rural and 23 urban). Seven of the 17 hospitals (University of Gondar Teaching Referral Hospital, Borumeda Hospital, Debre Markoss Referal Hospital, Woledia Hospital, Metema Hospital, Debre Tabor Hospital, and Debre Berhan Referral Hospital) were included in this study whereas the others were excluded because they have no MDR-TB treatment. In addition, patients that have no full history about their epidemiological, clinical and laboratory results were excluded from the study using exclusion criteria. The sample size was determined using at 95% CI with a prevalence of MDR-TB rate of 15% [18] and a margin error of 0.036. Then a total sample of 396 (377 and 5% contingency) MDR-TB patients were considered using simple random sampling methods. Further discussions on sampling are available at Cochran [19]. Thus, a total sample of 311 MDR-TB patients that fulfill the inclusion-exclusion criteria was considered applying simple random sampling methods. Data were collected by trained nurses under the supervision of investigators and the data quality had been checked for their completeness, consistency, and accuracy by investigators every day (Fig. 1).

### Measurements

The response variable in this study is defined to be the treatment period from the starting time of MDR-TB treatment up to the time of its cure. The event of interest was recovery from MDR-TB (1 = recovery and 0 = censored). The predictor variables that are included in this study were background characteristics of MDR-TB patients and history of epidemiological, clinical and laboratory results (Table 1). Data were entered and cleaned using SPSS-22 and analyzed using STATA-14.

**Table 1:** Predictors considered in the study

Variables	Categories
Sex	0 = Male
	1 = Female
Age	Continuous (year)
Residence	0 = urban,
	1 = Rural
Marital status	0 = Married,
	1 = Single,
	2 = Divorced,
	3 = widowed
HIV status	0 = HIV Negative
	1 = HIV Positive
Smoking history	1 = Smokers,
	0 = Non-smokers
Adherence based on Pill count (2 = good (100%), 1 = Fair (> = 80%) and 0 = poor (< 80%)	2 = Good
	1 = Fair
	0 = Poor
Comorbidities	1 = Yes
	0 = No
Previous drug use history	1 = yes
	0 = No
MDR-TB type	0 = pulmonary
	1 = Extra pulmonary
Baseline weight	Continuous (kg)
Occupation	0 = Employee
	1 = Farmer
	2 = Merchant

*HIV* human immunodeficiency virus*, MDR-TB* multidrug-resistant tuberculosis*, kg* kilo gram

### Statistical analysis

Survival analysis is the analysis of statistical data in which the outcome variable of interest is time until an event occurs. Survival data are almost always incomplete and called censoring that may be a right censor, left censoring and interval censoring. The most common are right censoring that happens when a subject follow-up times to terminate before the outcome of interest observed [20]. In any applied set, a survival data can summarize through life tables [21], Kaplan-Meier Survival functions [22] and median survival time [23, 24]. Besides estimating the survival functions, comparing two or more estimated survival curves is the most frequently used statistical tool of recent clinical research [25]. The simplest way of comparing the survival times obtained from two or more groups are the Kaplan-Meier curves and log-rank test [26–28]. However, to explore the relationship between the survival experience of individual and explanatory variables, an approach based on statistical modeling has been used [29]. Also used with a modeling approach to the analysis of survival data are the Cox Proportional Hazard [30], Accelerated Failure Time [30] and parametric shared frailty models [31].

### Accelerated failure time model

Parametric models are very applicable to analyze survival data; there are relatively few probability distributions of the survival time that can be used with these models. In Accelerated Failure Time (AFT) models, we measure the direct effect of the explanatory variables on the survival time, instead of a hazard. This allows an easier interpretation of the results because the parameters measure the effect of the correspondent covariate on the mean survival time. For AFT models it is common to use the log-linear representation:

$$ {Y}_i=\mathit{\log}{T}_i=\mu +{\beta}_1{x}_{1i}+\dots +{\beta}_p{x}_{pi}+\sigma {\varepsilon}_i, $$

where *logT*_*i*_ represents the log-transformed survival time, *μ* is the intercept and *σ* is the scale parameter, the *x*_1_, … , *x*_*p*_ are the explanatory variables with the coefficients *β* reflecting the effect that each explanatory variable have on the survival time and estimated by maximum likelihood method using a Newton-Raphson procedure and *ε*_*i*_ is the error term which is assumed to follow a specific distribution such as Weibull [32], log-normal [33], log-logistic [30] and gamma [34] among money.

### Parametric shared frailty models

In order to account for unobserved heterogeneity, the frailty term was first introduced by Hougaard in 1991 [35], which is an extension of proportional hazards. In a shared frailty model, lifetimes of a group of observations in the same cluster share the same level of frailty with each other [36] that the common frailty variance measures of dependence among lifetimes within a cluster.

Suppose that there are i clusters and each cluster i have n_i_ observations where ${\sum}_{i=1}^r{n}_i=n$ is the total sample size and t_ij_ = min(c_ij_, t^∗^_ij_) is the observed failure time of a right censoring scheme for k^th^ (k = 1, ..., n_i_) observation in i^th^ cluster and c_ij_ is the censoring time, where t^∗^_ij_ and c_ij_ are independent random variables [31]. Then the observed censoring indicator δ_ij_ is equal to 1 if t^∗^_ij_ < c_ij_, and 0 otherwise and conditional on frailty z_i_ (> 0) and X_ij_, the hazard function of i^th^ cluster has the form: *h*(*t*_*ij*_*X*_*ij*_, *z*_*i*_) = *z*_*i*_*h*_0_(*t*_*ij*_) exp(*β*^′^*X*_*ij*_), where *h*_0_(.) is the baseline hazard function, *X*_*ij*_ is a vector of observed predictors for the *k*^*th*^ observation and β is a vector of regression parameters.

The frailties, *z*_*i*_, are i.i.d. variables with the common probability density function *g*(*z*_*i*_). Various studies were done on the choice of continuous distribution of frailty random variables such as Gamma [34], inverse Gaussian [36], log-normal [33] and positive stable [37] and few studies done on the discrete distributions [38]. However, the Gamma distribution is the most common and widely used in literature for determining the frailty effect, which acts multiplicatively on the baseline hazard [31]. Due to its computational convenience, Gamma distribution of mean 1 and variance θ used as the frailty distribution for this study.

The probability density functions of one parameter Gamma distribution is given as:

$$ {f}_z(z)=\frac{{z_i}^{\frac{1}{\theta }}\exp \left(\frac{-{z}_i}{\theta}\right)}{\theta^{\frac{1}{\theta }}\ \Gamma \left(\frac{1}{\theta}\right)}\kern5.75em \theta >0 $$

The larger value of θ indicates the greater degree of heterogeneity among lifetimes within a cluster. Under the concept of gamma frailty, unconditional survival functions as cluster *i* obtained by integrating the conditional survival function of the Gamma distribution.

Once the parametric form of baseline hazard specified, the unconditional likelihood function can be easily derived [31, 39, 40] as:

$$ L\left(\psi, \theta, \beta \right)=\prod \limits_{i=1}^n\frac{\Gamma \left({D}_i+\raisebox{1ex}{$1$}\!\left/ \!\raisebox{-1ex}{$\theta $}\right.\right)\prod \limits_{k=1}^{n_i}{\left({h}_0\left({t}_{ij},\psi \right){e}^{\beta^{\prime }{X}_{ij}}\right)}^{\delta_{ij}}}{\theta^{\raisebox{1ex}{$1$}\!\left/ \!\raisebox{-1ex}{$\theta $}\right.}\Gamma \left(\raisebox{1ex}{$1$}\!\left/ \!\raisebox{-1ex}{$\theta $}\right.\right){\left(\raisebox{1ex}{$1$}\!\left/ \!\raisebox{-1ex}{$\theta $}\right.+\sum \limits_{k=1}^{n_i}{\Lambda}_0\left({t}_{ij},\psi \right){e}^{\beta^{\prime }{X}_{ij}}\right)}^{\raisebox{1ex}{$1$}\!\left/ \!\raisebox{-1ex}{$\theta $}\right.+{D}_i}} $$,

where *i* = 1, ..., *n* and *k* = 1, ..., *n*_*i*_, ψ is the vector of baseline hazard parameters, ${\Lambda}_0(.)=\underset{0}{\overset{t_{ij}}{\int }}{h}_0(s) ds$ is the common cumulative baseline hazard and β’s are the regression parameters that are estimated based on the marginal likelihood in which the frailties have been integrated out by averaging the conditional likelihood with respect to the frailty distribution [31, 40].

### Models comparison and diagnostics

There are several methods of model selection. But in this study, AIC [41] criteria were used to compare various candidate models and the model with the smallest AIC value is considered as a better fit [42]. After a model fitted, the adequacy of the fitted model needs to be assessed. The methods that involved the model checking for this study used evaluation of the Parametric Baselines and the Cox-Snell Residuals [43].

## Result

### Data exploratory

The study revealed that the recovery time of MDR-TB patients in Amhara region was 21 months with minimum and maximum recovery time of 17 and 25 months. Table 2 showed that out of the total MDR-TB patients, 110 (35.4%) were censored and 201 (64.6%) were cured of MDR-TB.

**Table 2:** Characteristics of MDR-TB patients in Amhara region, Ethiopia

Predictors	Labels	Status of Patients		
		Total	Cured/Event (%)	Censored
Sex	Female	133	79 (39.3)	54
	Male	178	122 (60.7)	56
Residence	Urban	167	106 (52.7)	61
	Rural	144	95 (47.3)	49
Treatment Center	Gondar	110	57 (28.4)	53
	Boromeda	48	33 (16.4)	15
	Debre Markos	46	32 (15.9)	14
	Woldeia	33	27 (33)	6
	Metema	30	20 (10)	10
	Debre Tabore	25	18 (9)	7
	Debre Berhan	19	14 (7)	5
Disease category	Pulmonary	262	194 (96.5)	68
	Extra Pulmonary	49	7 (3.7)	42
Drug using history	Yes	233	147 (73.1)	86
	No	78	54 (26.9)	24
Clinical	Complication	129	67 (29.9)	62
Complication	Not Complication	182	134 (70.1)	48
HIV status	HIV Positive	74	42 (20.9)	32
	HIV Negative	237	159 (79.1)	78
Smoking history	Yes	72	50 (57.1)	22
	No	239	151(24.9)	88
Comorbidities	Yes	27	18 (66.7)	9
	No	284	183 (64.4)	101
Adherence	Good	226	176 (77.9)	50
	Fair	39	19 (48.7)	20
	Poor	46	6 (13)	40
Occupation	0 = Employee	34	21(10.45)	13
	1 = Farmer	195	123 (61.12)	72
	2 = Merchant	82	57 (28.36)	25

**Fig. 2:**
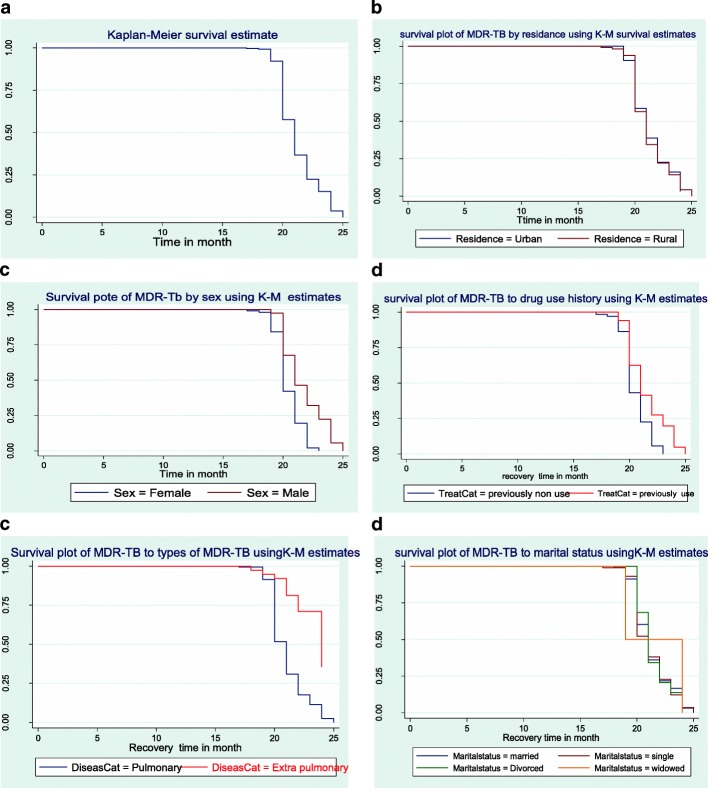
The K-M Survival function for the recovery time of MDR-TB patients in Amhara region by (**a**) overall (**b**) residence (**c**) sex (**d**) drug use history (**e**) type of MDR-TB (**f**) marital status

The Kaplan-Meier survival function is an important tool for analyzing censored data [31, 44]. The Kaplan-Meier estimator survival curve depicted the overall estimated survivor function and different groups of predictors. Clearly, the overall estimated survivor function showed that MDR-TB patients cured after the 20 months treatment. In addition, the survival ability of patients was the difference between sex, HIV status, smoking, and clinical complication whereas, between residence, marital status, education level, and occupation did not show a clear difference (Fig. 2a-f).

The observed difference in survival experiences in different patient groups was also assessed using the Long-rank and Breslaw test. Table 3 shows that there is a significant survival ability difference between sex, disease categories, drug use history, clinical complication, HIV status, adherence, smoking status, and comorbidity status at 5% significant level. In addition, sex with HIV and drug use history with the smoker is statistical significant interaction effect on MDR-TB patients.

**Table 3:** The Log-rank and Breslaw test of predictors for the recovery time of MDR-TB patients in Amhara region, Ethiopia

Covariates	Categories	Median	Log Rank test			Breslow test		
			Chi-square	df	*P*- value	Chi-square	Df	*P*- value
Sex	Female	20	33.18	1	< 0.001	27.17	1	< 0.001
	Male	21						
Marital status	Married	21	0.39	3	0.94	1.58	3	0.66
	Single	21						
	Divorce	21						
	Widowed	19						
Residence	Urban	21	0.05	1	0.94	0.05	1	0.82
	Rural	21						
Education	Not Educated	21	6.91	3	0.07	9.07	3	< 0.03
	Primary	21						
	Second	20						
	Above	20						
Disease category	Pulmonary	21	27.52	1	< 0.001	23.1	1	< 0.001
	Extra pull	24						
Drug using	Yes	21	15.44	1	< 0.001	11.67	1	< 0.001
History	No	22						
Clinical Complication	Yes	22	55.14	1	< 0.001	44.11	1	< 0.001
	No	20						
HIV status	Yes	22	23.03	1	< 0.001	12.05	1	< 0.001
	No	21						
Adherence	Good	21	37.59	2	< 0.001	21.77	1	< 0.001
	Fair	24						
	Poor	24						
Smoking status	Yes	23	38.38	1	< 0.001	19.77	1	< 0.001
	No	21						
Co morbidity status	Yes	21	45.65	1	< 0.001	29.7	1	< 0.001
	No	24						

### Accelerated failure time models

After checking the significance of categorical predictors by K-M survival function and the log-rank test, different AFT models were fitted. Among the Log-normal, Log-logistic, Exponential, Gamma and Weibull distributions, the Weibull AFT model was selected as the AIC and BIC were the smallest as showed in Table 4. Hence, the Weibull AFT model with a combination of covariates was used as the baseline hazard distribution of the parametric shared frailty model.

**Table 4:** Model selection based on IC from AFT models

IC	AFT models for baseline hazard distribution				
	Exponetial	Weibull	Log-normal	Log-logistic	Best model
AIC	496.83	−600.58	−598.52	− 561.97	Weibull
BIC	526.75	−566.92	− 564.86	− 528.31	Weibull

*AIC* Akaike’s Information Criteria*, BIC* Bayesian Information Criteria*, IC* Information Criteria

**Fig. 3:**
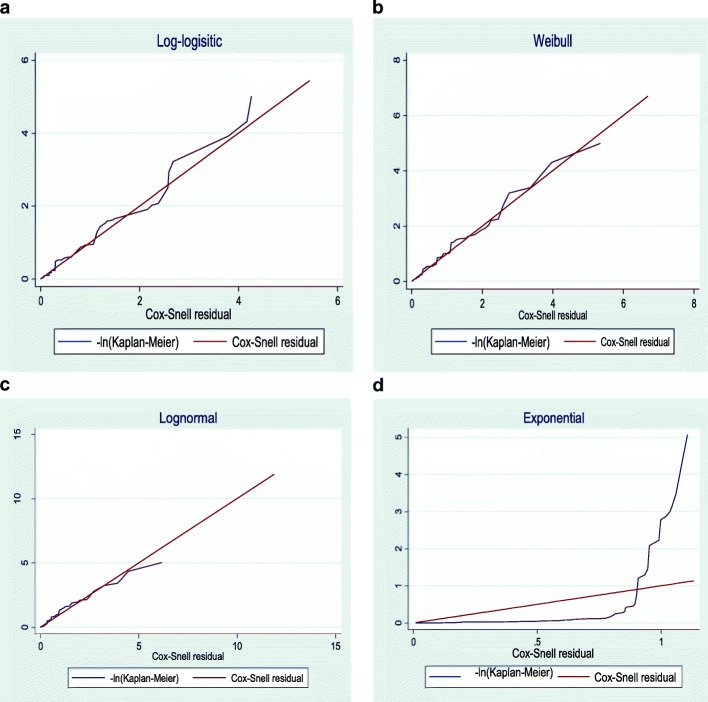
The Cox-Snell residual plots for (**a**) log-logistic (**b**) Weibull (**c**) lognormal (**d**) exponential distribution

The overall goodness of fit for the AFT model was checked by the Cox-Snell residual plots [43]. As Fig. 3 showed, the line related to the Cox-Snell residual of the Weibull AFT model was the nearest to 45^0^ straight lines of the origin when compared to that of exponential, lognormal and log-logistic models (Fig. 3a-d).

### Parametric shared frailty models

Gamma distribution is the most common and widely used in literature for determining the frailty effect [40, 45]. Accordingly, the Gamma frailty and Weibull Gamma shared frailty model was fitted to select the best model for this study using hospitals as random (frailty). The AIC for the Weibull gamma shared frailty (− 6003.79) was smaller than the AIC for the Weibull AFT (− 600.58) and Gamma shared frailty (− 592.53) models. The frailty for the selected model was estimated to be 1.2397 (chi-square = 34.07, df = 1, *p*-value = 0.0001) which indicated existence of unobserved heterogeneity between the hospitals and that the frailty component in the model was important. Hence the recovery time of MDR-TB patients in Amhara region, Ethiopia was carried out by the Weibull Gamma shared frailty model with hospitals as a clustering effect. Table 5 depicted the result of Weibull Gamma shared frailty model of parameter estimates, time ratio, standard error of estimates, z-value, *p*-values and 95% CI.

**Table 5:** Multivariable analysis using Weibull shared Gamma frailty model for the recovery time of MDR-TB patients in Amhara region, Ethiopia

Variables	Categories	Coeff.	*T*. *R*(ϕ)	Std Error	Z-value	*P* > |z|	95% CI for ϕ	
MDR-TB type	Extra pulmonary	0.05	1.055	0.013	4.34	< 0.001	1.03	1.08
	Pulmonary^+^		1					
Clinical Complication	Yes	0.04	1.041	0.008	5.23	< 0.001	1.025	1.056
	No^+^		1					
Adherence	Fair	0.02	1.021	0.019	1.09	0.28	0.984	1.059
	Poor	0.031	1.032	0.013	2.46	< 0.01	1.006	1.058
	Good^+^		1					
Co morbidities	Yes	0.078	1.081	0.015	5.69	< 0.001	1.053	1.111
	No^+^		1					
HIV status	Positive	−0.007	0.993	0.014	−0.51	0.613	0.966	1.021
	Negative^+^		1					
Sex	Male	0.028	1.029	0.007	4.02	< 0.001	1.015	1.043
	Female^+^		1					
Previous drug use history	Yes	−0.009	0.992	0.008	−1.06	0.291	0.976	1.007
	No^+^		1					
Smoking status	Smoker	−0.051	0.95	0.02	−2.43	0.015	0.9125	0.99
	Nonsmoker^+^		1					
Sex by HIV status	Male HIV positive	0.037	1.038	0.019	2.03	0.042	1.001	1.075
	Female HIV negative^+^		1					
Drug use history by smoker	Drug used with smoker	0.071	1.073	0.025	3.09	0.002	1.026	1.122
	Nondrug use with nonsmoker^+^		1					
Cons		2.99	19.879	0.136	438.53	< 0.001	19.615	20.146
Ln(*λ*)			3.722	0.106	35.02	< 0.001	3.514	3.931
Ln(*θ*)			−3.986	1.775	−2.25	0.03	−7.464	−0.508
*λ*			41.364	4.397			33.585	50.946
*γ*			0.024	0.003			0.02	0.03
*θ*			1.24	0.3608			0.695	2.212

*γ* Shape parameter*, λ* Scale parameter*,θ* Frailty parameter, ^*+*^ reference category*, CI* Confidence interval*, Std Error* Standard Error, *ϕ* Time ratio*:* the ratio for groups of median time to recovery

After adjusting other covariates, the hazard of attaining immunological recovery of patients that had extrapulmonary MDR-TB (ϕ=1.0546, 95% CI: 1.0296–1.0802), clinical complication (ϕ=1.0405, 95% CI: 1.025–1.056), comorbidity (ϕ=1.0814, 95% CI: 1.0526–1.1109), male (ϕ=1.037287, 95% CL: 1.0146–1.043) and having poor adherence (ϕ = 1.0316, 95%CI: 1.0063–1.0575) were 5.46, 4.05, 8.14, 3.73, and 3.16% higher than that of the counter groups..

## Discussion

In this study, the 311 MDR-TB patients in Amhara region, Ethiopia were assessed, of which, 110 (35.4%) were censored and 201 (64.6%) were cured of MDR-TB. Overall, the median recovery time of MDR-TB patients in Amhara region was 21 months, which means that the recovery time of patients is within the recommended treatment interval of 18 to 24 months or longer [10] given close monitoring of patients while taking these drugs.

The Weibull AFT model had the smallest AIC compared to that of Log-normal, Log-logistic, Exponential, Gamma and gamma AFT models. After selected the Weibull AFT model, Weibull Gamma shared frailty model was well fitted to the data onto Weibull AFT, Gamma frailty and Weibull Gamma shared frailty models. This is because of the model lifetime, Weibull distribution is mostly used in the literature as the Hazard rate for Weibull distribution is a monotone function [31, 46–48] and the Newton–Raphson procedure is used to maximize the best-unbiased estimator for the model parameters [39]. Thus, the MDR-TB patients in Amhara region, Ethiopia were analyzed using the Weibull gamma shared frailty model. This study identified a number of factors associated with the recovery time of MDR-TB patients in Amhara region, Ethiopia taking the hospital’s clustering effect. The clustering effect was significant (*p*-value < 0.001) in Weibull-gamma shared frailty model that indicates heterogeneity between hospitals and patients within the same hospital share similar risk factors on cure time. This showed that the correlation between hospitals cannot be ignored and the clustering effect was important in modeling the hazard function. Exploring patients increased insight into patient handling, and gave valuable information about providing attention and quality services to patients. The study showed that extrapulmonary MDR-TB patients had longer recovery time than that of seamier pulmonary MDR-TB patients in Amhara region, Ethiopia, and it was supported by Parmar et al study [49].

Male MDR-TB patients were associated with a high likelihood of experiencing unsuccessful treatment outcomes. Several other studies have similarly shown that male MDR-TB patients tend to have longer recovery time [49–51]. This means that male MDR-TB patients were experiencing longer recovery time than that of female MDR-TB patients. This might be related to higher tendencies towards alcohol and drug abuse, and interruption of their medication as a male has high economical consequences than that of females, while the biochemical, behavioral and socioeconomic determinants of males need further study. The MDR-TB patients with co-morbidity and clinical complication also experienced longer recovery time than that of the control groups. This result is in line with the previous findings in Ethiopia [52, 53] and in India [49]. Furthermore, TB with clinical complication/co-infection often associates with poor response to TB-medications due to nutrition absorption of anti-tuberculosis drugs, overlap toxicity in ART patients and/or due to the risk of immune reconstitution inflammatory syndrome [54]. Unlike this finding HIV status was not a statistically significant predictor variable. This difference may arise from the sample size difference, model difference, as well as the nature of the population. The results of this study revealed that adherence was a significant factor of recovery time of MDR-TB patients in Amhara Region, Ethiopia. These results showed that MDR-TB patients with poor adherence had longer recovery time than MDR-TB patients who had good adherence. This finding was similar with a study [49, 55, 56]. The results of this study suggested that the interaction of smoking status with previous drug use history was a significant predictive factor of time to cure in the Amhara region. This shows that smokers with previously drug user history take more time to cure than patients having no previous history of smoking. This was consistent with the study done by Kuaban et al [57].

### Limitations

The limitations of this study were the small sample of participants. There are also a variety of predictors, an assessment of which was beyond the scope of this study, but that might influence the MDR-TB patients. Thus, researchers may consider other predictors to identify important predictors of BDR-TB patients that delay cured time.

## Conclusion

This study aimed to investigate the major factors that affect recovery time of MDR-TB patients in Amhara region, Ethiopia. The study revealed that the median recovery time of MDR-TB patients in Amhara region is 21 months, and that close monitory of patients is required while they take these drugs. In general, it has been concluded that this has the undesirable consequences of economic, social and political weaknesses, shortage of health facilities and access to hospitals in the region. Thus, the regional and federal Government of Ethiopia need to take immediate steps to address the causes of long recovery time of MDR-TB patients in Amhara region, Ethiopia. This may for example involve, strengthening the counseling of patients and family members as well as the use of digital tools in monitoring treatment adherence to decrease the likelihood of recovery time. In addition, there should be robust early case detection and proper treatment of drug-susceptible MDR-TB to shorten the recovery time of MDR-TB in accordance with WHO guidelines.

## Abbreviations

ADIS: Acquired Deficiency Immune Syndrome
AFT: Accelerated Failure Time
AIC: Akaki Information Criteria
ART: Antiretroviral therapy
CI: Confidence Interval
CSA: Central statistical agency
HIV: Human Immunodeficiency Virus
MDR-TB: Multidrug resistance tuberculosis
PH: Proportional Hazard
TB: Tuberculosis
WHO: World Health Organization
XDR: Extensively drug-resistant

## References

[1] WHO (2017). Global tuberculosis report 2017.

[2] WHO (2010). Multidrug and extensively drug-resistant TB.

[3] Faustini A, Hall AJ, Perucci CA (2006). Risk factors for multidrug resistant tuberculosis in Europe: a systematic review. Thorax.

[4] Kundu D, Sharma N, Chadha S, Laokri S, Awungafac G, Jiang L, Asaria M (2018). Analysis of multi drug resistant tuberculosis (MDR-TB) financial protection policy: MDR-TB health insurance schemes, in Chhattisgarh state, India. Health Econ Rev.

[5] Singh JA, Upshur R, Padayatchi N (2007). XDR-TB in South Africa: no time for denial or complacency. PLoS Med.

[6] Koul A, Arnoult E, Lounis N, Guillemont J, Andries K (2011). The challenge of new drug discovery for tuberculosis. Nature.

[7] Assefa D, Seyoum B, Oljira L (2017). Determinants of multidrug-resistant tuberculosis in Addis Ababa, Ethiopia. Infect Drug Resist.

[8] Eldholm V, Balloux F (2016). Antimicrobial resistance in mycobacterium tuberculosis: the odd one out. Trends Microbiol.

[9] Getachew T, Bayray A, Weldearegay B (2013). Survival and predictors of mortality among patients under multi-drug resistant tuberculosis treatment in ethiopia: st. Peter's specialized tuberculosis hospital, ethiopia. Int J Pharm Sci Res.

[10] Falzon D, Schünemann HJ, Harausz E, González-Angulo L, Lienhardt C, Jaramillo E, Weyer K (2017). World Health Organization treatment guidelines for drug-resistant tuberculosis, 2016 update. Eur Respir J.

[11] Espinal MA, Laszlo A, Simonsen L, Boulahbal F, Kim SJ, Reniero A, Hoffner S, Rieder HL, Binkin N, Dye C (2001). Global trends in resistance to antituberculosis drugs. N Engl J Med.

[12] Tyrrell F, Stafford C, Yakrus M, Youngblood M, Hill A, Johnston S (2017). Trends in testing for mycobacterium tuberculosis complex from US public health laboratories, 2009–2013. Public Health Rep.

[13] Tacconelli E, Cataldo M, Dancer S, De Angelis G, Falcone M, Frank U, Kahlmeter G, Pan A, Petrosillo N, Rodríguez-Baño J (2014). ESCMID guidelines for the management of the infection control measures to reduce transmission of multidrug-resistant gram-negative bacteria in hospitalized patients. Clin Microbiol Infect.

[14] Alrabiah K, Al Alola S, Al Banyan E, Al Shaalan M, Al Johani S (2016). Characteristics and risk factors of hospital acquired–methicillin-resistant Staphylococcus aureus (HA-MRSA) infection of pediatric patients in a tertiary care hospital in Riyadh, Saudi Arabia. Int J Pediatr Adolesc Medi.

[15] Tarai B, Das P, Kumar D (2013). Recurrent challenges for clinicians: emergence of methicillin-resistant Staphylococcus aureus, vancomycin resistance, and current treatment options. J Lab Phys.

[16] Fair RJ, Tor Y. Antibiotics and bacterial resistance in the 21st century. Perspect Med Chem. 2014;6:25–64. 10.4137/PMC.S14459. 10.4137/PMC.S14459 PMC4159373 25232278

[17] Agerie NW. Determinants of smallholder rural farm households’ participation in small scale irrigation and its effect on income in North Gondar zone: a cross-sectional approach (evidence from Dembia Woreda). Ethiopia: Mekelle University; 2013.

[18] Nigus D, Lingerew W, Beyene B, Tamiru A, Lemma M, Melaku M (2014). Prevalence of multi drug resistant tuberculosis among presumptive multi drug resistant tuberculosis cases in Amhara National Regional State, Ethiopia. J Mycobac Dis.

[19] Cochran WG (1977). Sampling Techniques.

[20] Fan J. Local polynomial modelling and its applications: monographs on statistics and applied probability 66. New York: Routledge; 2018.

[21] van der Meulen A (2012). Life tables and survival analysis.

[22] Dabrowska DM. Kaplan-Meier estimate on the plane. Ann Stat. 1988;16(4):1475–89.

[23] Brookmeyer R (2014). Median survival time.

[24] Reid N (1981). Estimating the median survival time. Biometrika.

[25] Brueffer C, Vallon-Christersson J, Grabau D, Ehinger A, Häkkinen J, Hegardt C, Malina J, Chen Y, Bendahl P-O, Manjer J (2018). Clinical value of RNA sequencing–based classifiers for prediction of the five conventional breast Cancer biomarkers: a report from the population-based multicenter Sweden Cancerome analysis network—breast initiative. JCO Precis Oncol.

[26] Harrington DP, Fleming TR (1982). A class of rank test procedures for censored survival data. Biometrika.

[27] Xie J, Liu C (2005). Adjusted Kaplan–Meier estimator and log-rank test with inverse probability of treatment weighting for survival data. Stat Med.

[28] Kleinbaum DG, Klein M. Kaplan-Meier survival curves and the log-rank test. In: Survival analysis. New York: Springer; 2012. p. 55–96.

[29] Collett D. Modelling survival data in medical research. New York: Chapman and Hall/CRC; 2015.

[30] Cox DR. Regression models and life-tables. In: Breakthroughs in statistics. New York: Springer; 1992. p. 527–41.

[31] Gutierrez RG (2002). Parametric frailty and shared frailty survival models. Stata J.

[32] Pike M (1966). A method of analysis of a certain class of experiments in carcinogenesis. Biometrics.

[33] Bennett S (1983). Analysis of survival data by the proportional odds model. Stat Med.

[34] Van den Berg GJ. Duration models: specification, identification and multiple durations. In: Handbook of econometrics, vol. 5. Germany: Elsevier; 2001. p. 3381–460.

[35] Hougaard P (1991). Modelling heterogeneity in survival data. J Appl Probab.

[36] Aalen O, Borgan O, Gjessing H. Survival and event history analysis: a process point of view. New York: Springer Science & Business Media; 2008.

[37] Hougaard P (1986). Survival models for heterogeneous populations derived from stable distributions. Biometrika.

[38] Caroni C, Crowder M, Kimber A (2010). Proportional hazards models with discrete frailty. Lifetime Data Anal.

[39] Duchateau L, Janssen P. The frailty Model. New York: Springer-Verlega; 2008.

[40] Askin OE, Inan D, Buyuklu AH. Parameter Estimation of Shared Frailty Models Based on Particle Swarm Optimization. Int J Stat Probab. 2017;6(1):48–58.

[41] Akaike H (1974). A new look at the statistical model identification. IEEE Trans Autom Control.

[42] Munda M, Rotolo F, Legrand C (2012). Parfm: parametric frailty models in R. J Stat Softw.

[43] Cox DR, Snell EJ. A general definition of residuals. J R Stat Soc Ser B Methodol. 1968;30(2):248–75.

[44] Miller Jr, R.G. Survival Analysis. Vol. 66. Chichaster: Wily; 2011.

[45] Wienke A. Frailty models in survival analysis. New York: Chapman and Hall/CRC; 2010.

[46] Swain PK, Grover G (2016). Accelerated failure time shared frailty models: application to HIV/AIDS patients on anti-retroviral therapy in Delhi, India. Turkiye Klinikleri J Biostat.

[47] Sahu SK, Dey DK, Aslanidou H, Sinha D (1997). A Weibull regression model with gamma frailties for multivariate survival data. Lifetime Data Anal.

[48] Ibrahim JG, Chen M-H, Sinha D. Bayesian survival analysis. New York: Springer Science & Business Media; 2001.

[49] Parmar MM, Sachdeva KS, Dewan PK, Rade K, Nair SA, Pant R, Khaparde SD (2018). Unacceptable treatment outcomes and associated factors among India's initial cohorts of multidrug-resistant tuberculosis (MDR-TB) patients under the revised national TB control programme (2007–2011): evidence leading to policy enhancement. PLoS One.

[50] Hamusse SD, Demissie M, Teshome D, Lindtjørn B (2014). Fifteen-year trend in treatment outcomes among patients with pulmonary smear-positive tuberculosis and its determinants in Arsi zone, Central Ethiopia. Glob Health Action.

[51] Brust JCMGN, Carrara H, Osburn G, Padayatchi N (2010). High treatment failure and default rates for patients with multidrug-resistant tuberculosis in KwaZulu-Natal, South Africa, 2000–2003. Int J Tuberc Lung Dis.

[52] Girum T, Tariku Y, Dessu S. Survival status and treatment outcome of multidrug resistant tuberculosis (MDR-TB) among patients treated in treatment initiation centers (TIC) in South Ethiopia: a retrospective cohort study. Ann Med Health Sci Res. 2017;7(5):331–36.

[53] Mitku AA, Dessie ZG, Muluneh EK, Workie DL (2016). Prevalence and associated factors of TB/HIV co-infection among HIV infected patients in Amhara region, Ethiopia. Afr Health Sci.

[54] Rabie H, Decloedt EH, Garcia-Prats AJ, Cotton MF, Frigati L, Lallemant M, Hesseling A, Schaaf HS (2017). Antiretroviral treatment in HIV-infected children who require a rifamycin-containing regimen for tuberculosis. Expert Opin Pharmacother.

[55] Cox HS, McDermid C, Azevedo V, Muller O, Coetzee D, Simpson J, Barnard M, Coetzee G, van Cutsem G, Goemaere E (2010). Epidemic levels of drug resistant tuberculosis (MDR and XDR-TB) in a high HIV prevalence setting in Khayelitsha, South Africa. PLoS One.

[56] Deshmukh RDDD, Sachdeva KS, Sreenivas A, Kumar AMV, Satyanarayana S, et al. Patient and provider reported reasons for lost to follow up in MDRTB treatment: a qualitative study from a drug resistant TB Centre in India. PLoS One. 2015;10(8):1–11. 10.1371/journal.pone.0135802 PMC4547708 26301748

[57] Kuaban C, Noeske J, Rieder H, Aït-Khaled N, Abena Foe J, Trébucq A (2015). High effectiveness of a 12-month regimen for MDR-TB patients in Cameroon. Int J Tuberc Lung Dis.

